# Reverse Genetics in *Candida albicans* Predicts ARF Cycling Is Essential for Drug Resistance and Virulence

**DOI:** 10.1371/journal.ppat.1000753

**Published:** 2010-02-05

**Authors:** Elias Epp, Ghyslaine Vanier, Doreen Harcus, Anna Y. Lee, Gregor Jansen, Michael Hallett, Don C. Sheppard, David Y. Thomas, Carol A. Munro, Alaka Mullick, Malcolm Whiteway

**Affiliations:** 1 Biotechnology Research Institute, National Research Council of Canada, Montréal, Québec, Canada; 2 Department of Biology, McGill University, Montréal, Québec, Canada; 3 Department of Microbiology and Immunology, McGill University, Montréal, Québec, Canada; 4 McGill Centre for Bioinformatics, McGill University, Montréal, Québec, Canada; 5 Department of Biochemistry, McGill University, Montréal, Québec, Canada; 6 Département de Microbiologie et Immunologie, l'Université de Montréal, Montréal, Québec, Canada; 7 School of Medical Sciences, University of Aberdeen, Aberdeen, United Kingdom; Carnegie Mellon University, United States of America

## Abstract

*Candida albicans*, the major fungal pathogen of humans, causes life-threatening infections in immunocompromised individuals. Due to limited available therapy options, this can frequently lead to therapy failure and emergence of drug resistance. To improve current treatment strategies, we have combined comprehensive chemical-genomic screening in *Saccharomyces cerevisiae* and validation in *C. albicans* with the goal of identifying compounds that can couple with the fungistatic drug fluconazole to make it fungicidal. Among the genes identified in the yeast screen, we found that only *AGE3*, which codes for an ADP-ribosylation factor GTPase activating effector protein, abrogates fluconazole tolerance in *C. albicans*. The *age3* mutant was more sensitive to other sterols and cell wall inhibitors, including caspofungin. The deletion of *AGE3* in drug resistant clinical isolates and in constitutively active calcineurin signaling mutants restored fluconazole sensitivity. We confirmed chemically the *AGE3*-dependent drug sensitivity by showing a potent fungicidal synergy between fluconazole and brefeldin A (an inhibitor of the guanine nucleotide exchange factor for ADP ribosylation factors) in wild type *C. albicans* as well as in drug resistant clinical isolates. Addition of calcineurin inhibitors to the fluconazole/brefeldin A combination only initially improved pathogen killing. Brefeldin A synergized with different drugs in non-*albicans Candida* species as well as *Aspergillus fumigatus*. Microarray studies showed that core transcriptional responses to two different drug classes are not significantly altered in *age3* mutants. The therapeutic potential of inhibiting ARF activities was demonstrated by *in vivo* studies that showed *age3* mutants are avirulent in wild type mice, attenuated in virulence in immunocompromised mice and that fluconazole treatment was significantly more efficacious when ARF signaling was genetically compromised. This work describes a new, widely conserved, broad-spectrum mechanism involved in fungal drug resistance and virulence and offers a potential route for single or improved combination therapies.

## Author Summary

*Candida albicans* is a fungus that normally resides as part of the microflora in the human gut. *Candida* species can cause superficial infections like thrush in the healthy human population and life-threatening invasive infections in immunocompromised patients. Fungal infections are often treated with azole drugs, but due to the fungistatic nature of these agents, *C. albicans* can develop drug resistance, leading to therapy failure. To improve the action of azoles and convert them into fungicidal drugs, we first systematically analyzed the genetic requirements for tolerance to one such azole drug, fluconazole. We show, both genetically and pharmacologically, that components of the ARF cycling machinery are critical in mediating both azole and echinocandin tolerance in *C. albicans* as well as several other pathogenic *Candida* species and in the pathogenic mold *Aspergillus fumigatus*. We highlight the importance of ARF cycling in drug resistance by showing that genetic compromise of ARF functions overrides common drug resistance mechanisms in clinical samples and other key regulators of azole/echinocandin tolerance. We validated the therapeutic potential of ARF cycling in two mouse models and provide evidence that drug treatment is more efficacious when ARF activities are genetically compromised. Our study demonstrates a new mechanism involved in two important aspects of the biology of human fungal pathogens and provides a potential route for improved antifungal therapies.

## Introduction

Invasive fungal infections pose a serious health risk to hospitalized patients worldwide. Particularly affected are immunocompromised individuals with cancer or AIDS, people undergoing organ and hematopoietic stem cell transplantation (HSCT), and those receiving immunosuppressive therapy or implantable prosthetic devices [1],[2]. The growing population of these at-risk groups is reflected in an increase in invasive fungal infection over the last three decades [3]. Annual treatment costs for fungal therapies reach $2.6 billion in the US alone [4]. Despite available therapy options mortality rates approaching 30–50% (*Candida* species) and 30–80% (*Aspergillus* species) remain high [5],[6].

*Candida* and *Aspergillus* species together account for ∼70% of all invasive fungal infections, with *Candida albicans* and *Aspergillus fumigatus* predominating [7],[8],[9]. Currently, three classes of antifungal drugs are suitable for treatment of systemic infections caused by these fungi: polyenes (most notably amphotericin B) and azoles (e.g. fluconazole, FCZ) have been applied for decades, while the echinocandins (e.g. caspofungin, CF) represent a new class of antifungal that has entered treatment regimes over the past 10 years [10],[11]. While these therapy options can be effective, they also exhibit several shortcomings. First, current antifungals target a very limited number of biological processes. The majority of available drugs target ergosterol (polyenes) or inhibit lanosterol 14α-demethylase (azoles), resulting in the accumulation of toxic sterol intermediates that disrupt membrane integrity and lead to membrane stress. Because ergosterol, the major sterol in fungal cell membranes, is analogous to the mammalian lipid cholesterol, this strategy, particularly when amphotericin B is applied, can be problematic due to host toxicity [10]. Another complication of current antifungal strategies is that available drugs each possess a different spectrum of antifungal activities. For instance, azoles are typically fungistatic against pathogenic yeasts such as *Candida* species, but fungicidal against molds (*Aspergillus* species). CF, on the other hand, is fungicidal against yeasts and fungistatic against molds [12]. Finally, and most importantly, the small number of treatment options available has resulted in widespread drug resistance in pathogenic species. For each of the three major classes of antifungals (polyenes, azoles, echinocandins) isolation of drug-resistant clinical strains has been reported [11],[12],[13]; azole-resistant *Candida*, in particular, is now common among isolates from HIV-positive patients [14]. Developing new antifungal strategies, therefore, remains a pressing need.

One approach to satisfy this need is through combination antifungal therapy, where two (or more) agents combined are significantly more efficacious compared to either agent alone. This approach has recently been validated in a randomized, placebo-controlled trial, where approved antifungals were combined with immune regulatory agents [15]. Results from this study suggested that combining ergosterol inhibitors with a recombinant human monoclonal antibody against heat-shock 90 protein (HSP90) showed increased therapeutic benefits compared to monotherapy against *Candida* infection. Although the precise mechanisms involved remain elusive [16], extensive experiments have further established the benefits of such combinatorial approaches. For instance, a potent synergy resulted when inhibitors of HSP90 (geldanamycin, radicicol) or inhibitors of HSP90's key client protein, calcineurin (cyclosporin A (CsA), FK506) were combined either with azoles or echinocandins against *C. albicans* [10],[16],[17],[18],[19],[20],[21]. Similarly, pharmacological compromise of HSP90/calcineurin-signaling enhanced the efficaciousness of echinocandin treatment against *A. fumigatus in vitro* as well as in insect and mouse infection models [16],[17]. Although these examples clearly demonstrate the potential for combination antifungal therapy, human host toxicity associated with inhibition of HSP90 or suppression of the human immune system by CsA/FK506 currently precludes the use of such inhibitors in the clinic [16],[22]. While a non-immunosuppressive FK506 analogue (L-685, 818) has been identified, proprietary restrictions have currently prevented further testing [22]. Therefore, identification of new antifungal targets for optimal fungal killing remains a priority.

One of the challenges of finding new antifungal targets in *C. albicans* is the lack of sophisticated screening technologies often employed with, for example, *Saccharomyces cerevisiae*. Various large-scale chemical-genomic drug screening methods are now well established in *S. cerevisiae*, and have been effective for elucidating drug targets or revealing insights into the modes of action of bioactive compounds [23],[24],[25],[26],[27]. Similar approaches have only recently been applied directly to fungal pathogens [28]. Using *S. cerevisiae* as a model, we previously performed chemical-genomics to systematically analyze the genetic requirements to survive FCZ treatment [29]. In that work, we identified 22 genes that become essential for *S. cerevisiae* survival in the presence of FCZ.

Here, we expanded that work with the aim of identifying synergistic drug interactions that render FCZ fungicidal in *C. albicans*. To this end, we validated the *S. cerevisiae* FCZ-cidal gene set [29] in *C. albicans*. From 22 predicted genes, we found that only one gene, *AGE3*, mediated FCZ tolerance in the pathogen. We further show that both genetic and pharmacological compromise of ARF (ADP ribosylation factor) activities, a process that depends on Age3p, creates sensitivity to all three classes of antifungals used in clinics (polyenes, azoles, echinocandins), overrides clinical drug resistance and the calcineurin pathway, synergizes with fungistatic drugs against the two major pathogenic fungal species (*C. albicans* and *A. fumigatus*) and modifies fungal virulence in two established mouse models of candidiasis. Given that drug treatment in mice was significantly more efficacious when ARF activity was genetically compromised, this demonstrates that targeting ARF signaling has potential for antifungal therapies.

## Results

### Age3p mediates azole tolerance and sensitivity to cell wall inhibitors in *C. albicans*

To identify genes that become essential for survival in the presence of FCZ, we previously screened the non-essential *S. cerevisiae* knock-out collection (about 4900 strains) and identified 22 mutants that showed a robust FCZ-cidal phenotype [29]. BLAST searches identified *C. albicans* homologs for 21 of those *S. cerevisiae* genes. *YDR532c* appears to be the only gene that lacks a clear *C. albicans* homolog (Table S1). Recreating knockout or transposon insertion mutations of the 21 candidate genes in *C. albicans*, we found that four mutants (*bem2*, *sac6*, *srb8* and *ssn3*) showed FCZ sensitivity comparable to WT (Table S2, Figure S1). Twelve *C. albicans* mutants (57%) showed increased FCZ sensitivity, but all of these mutants could still resume growth when incubated for extended time in the presence of FCZ. Four genes (*GCN5*, *NGG1*, *ERG11* and *NUP84*) were linked to a slightly resistant FCZ phenotype. Only one mutant, *age3* (*ORF19.3683*), showed the FCZ sensitive phenotype predicted from the yeast screen. We therefore focused further investigation on *AGE3*.

**Figure 1:**
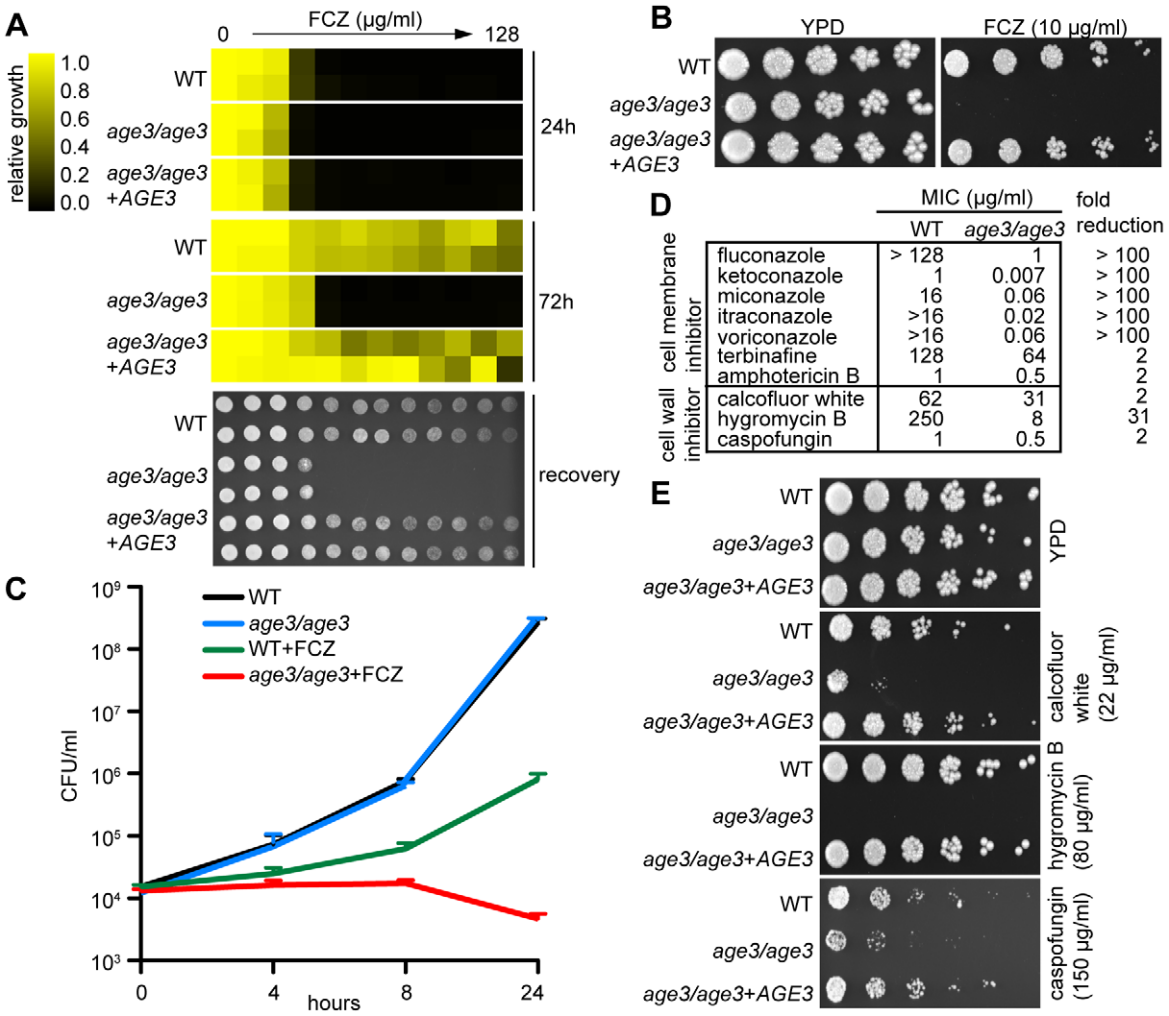
Age3p plays a major role in azole tolerance in *C. albicans*. (A) Minimal Inhibitory Concentration (MIC) assay in rich YPD media showing that *age3* cells are initially almost equally sensitive to FCZ compared to WT and revertant strains (24 hours reading, top), but fail to grow above this MIC threshold after prolonged incubation (72 hours reading, middle). MIC assays with two-fold serially diluted drug concentrations were done in duplicate and optical densities were normalized to drug-free control wells (see color bar). After 72 hours of incubation, 2 µl of each well of the MIC assay was spotted on fresh YPD media to assess the extent to which cells recover from the drug treatments (bottom). YPD recovery plates were incubated for 24 hours at 30°C. (B) FCZ sensitivity assayed on solid YPD media. No *age3* colonies grew on YPD plates containing 24 hours-supra-MIC concentrations of FCZ. Overnight cultures were adjusted to OD_600_ of 0.1, and then serially diluted four-fold, before 2 µl were spotted on plates. Plates were incubated for 48 hours at 30°C. (C) Time-kill curves in YPD media confirming that knocking out *AGE3* abrogates tolerance to FCZ. The number of viable *age3* cells decreases slightly over time, while growth of WT cells in the presence of FCZ still occurs. FCZ was used at 10 µg/ml. Shown is the average of two independent experiments plus SD values. Note that *age3* cells grow as efficiently as WT cells in the absence of drugs. (D) MIC assays in YPD media shows that *age3* mutants are extremely sensitive to numerous azoles after 48 hours and mildly more sensitive to non-azole ergosterol inhibitors (terbinafine, amphotericin B) as well as cell wall inhibitors when compared to WT cells. Fold reduction represents the ratio of the MIC value for WT over the MIC value of the *age3* mutant. (E) *age3* cells show differential sensitivity to different cell wall inhibitors on YPD media plates. The assay was done as described in (B).

We validated the FCZ sensitivity of *age3* cells by three different assays. When tested in a minimal inhibitory concentration (MIC) assay, the *age3* mutant initially showed similar drug sensitivity as WT and revertant strains at 24 hours. Since FCZ on its own is fungistatic, however, WT cells demonstrated robust growth above the initial MIC point after prolonged incubation (72 hours), a feature referred to as tolerance [19]. In contrast, *age3* cells did not resume growth above the 24 hours MIC point, indicating that *age3* mutants lost tolerance to FCZ (Figure 1A, Table S2). These results were confirmed visually by growth on solid rich media in the presence of FCZ (Figure 1B). We further characterized the FCZ sensitivity of *age3* cells by time-kill curves. Under FCZ treatment, the number of viable *age3* cells slightly decreased over time, while growth of WT cells in the presence of FCZ continued (Figure 1C).

We then tested the *age3* mutant against a variety of antifungals to gauge the specificity of the mutation. We included various compounds, including second-generation azoles (voriconazole), non-azole ergosterol inhibitors (terbinafine) and other membrane-targeting drugs (amphotericin B). We found that *age3* cells showed a generalized increased sensitivity to these compounds (Figure 1D). Among all cell-membrane drugs tested, the azoles caused by far the most significant enhancement in sensitivity in the *age3* mutant.

In order to determine the effect of deleting *AGE3* on the integrity of the cell wall, we tested the *age3* mutant for sensitivity to a variety of cell wall perturbing agents and other agents whose effect have been linked to altered cell wall and glycosylation. The *age3* mutant was slightly more sensitive to the β-1,3 glucan synthase inhibitor CF. Similarly, *age3* cells were slightly more sensitive to calcofluor white, a phenotype that is usually associated with altered chitin structures along the cell wall [30]. More remarkably, *age3* cells were extremely sensitive to hygromycin B, a phenotype usually seen in glycosylation mutants [31],[32] (Figure 1D, 1E). No change in sensitivity was observed in the presence of other agents such as caffeine, cycloheximide, menadione, nocodazole, rapamycin, 5-FC and wortmannin (data not shown). Together, these data suggest that while *AGE3* plays a major role during membrane stress in *C. albicans*, its influence on the integrity of the cell wall remains somewhat less clear (see discussion).

### Deleting *AGE3* overrides clinical drug resistance and the calcineurin pathway

**Figure 2:**
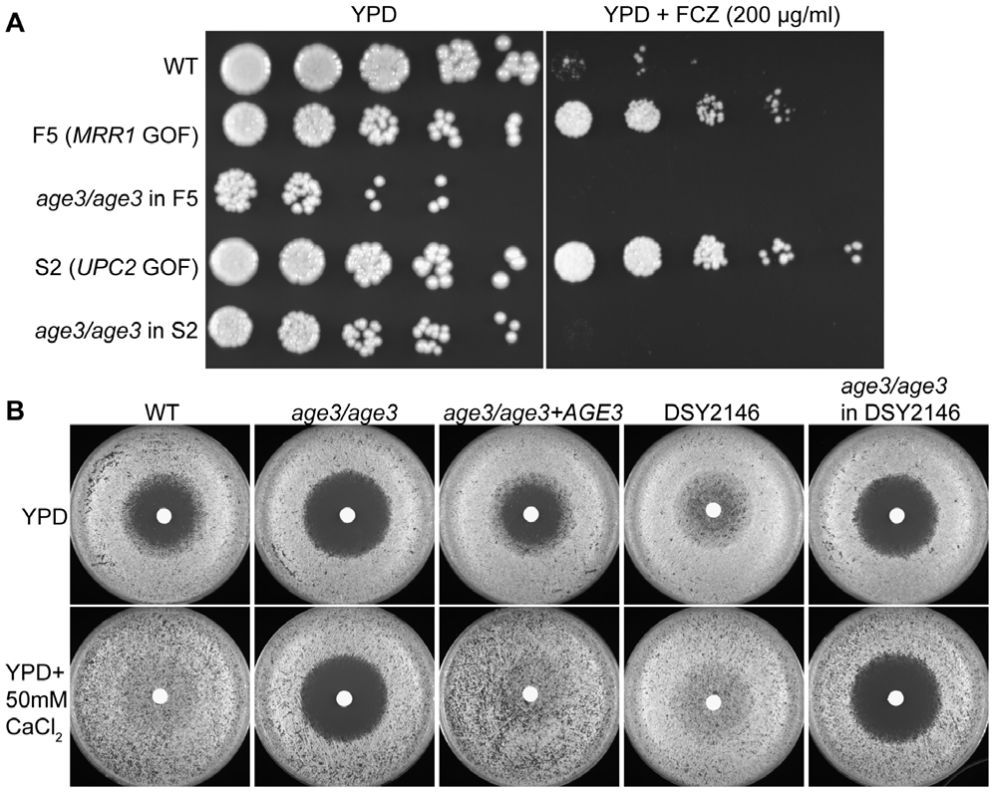
Deleting *AGE3* overrides clinical drug resistance and the calcineurin pathway. (A) When *AGE3* is knocked out in drug resistant clinical isolates F5 and S2, FCZ sensitivity is restored even below WT levels on solid YPD media. The assay was performed and analyzed as described in Figure 1B except plates were photographed after 24 hours. GOF = gain of function. (B) Calcineurin signaling stimulated either by extracellular CaCl_2_ or by a constitutively active mutation in strain DSY2146 leads to FCZ resistance. *age3* mutants do not respond to extracellular CaCl_2_, while knocking out *AGE3* in strain DSY2146 restored FCZ sensitivity. Disc diffusion assays were done by plating 2×10^5^ cells on YPD plates followed by applying discs containing 50 mg of FCZ to the surface of agar. Plates were incubated for 24 hours at 30°C.

Among the most commonly encountered resistance mechanisms in drug treated clinical *C. albicans* isolates are over-expression of drug pumps or alterations in sterol biosynthesis [13],[33],[34]. To test whether such common mechanisms of drug resistance are still effective in the absence of *AGE3*, we deleted *AGE3* in two FCZ-resistant clinical strains. The strain F5 carries a mutation in the transcription factor *MRR1*, which leads to constitutive over-expression of drug pumps. The strain S2 carries a mutation in the transcription factor *UPC2*, which causes up-regulation of ergosterol biosynthesis genes [35],[36]. Figure 2A shows that deleting *AGE3* in strains F5 and S2 restored FCZ sensitivity even below WT levels, suggesting that loss of *AGE3* abrogates FCZ-resistance in these clinical isolates.

Because *age3* mutants lost tolerance to FCZ and because the calcineurin pathway is known to mediate FCZ-tolerance in WT as well as drug resistant clinical isolates [10],[19], we tested whether constitutive calcineurin signaling could reverse the *AGE3*-dependent FCZ sensitivity. The calcineurin pathway can be activated by addition of extracellular CaCl_2_ [19]. *C. albicans* WT became resistant to FCZ after only 24 hours of growth in the presence of extracellular CaCl_2_, while this rescue was not observed in the absence of *AGE3* (Figure 2B). To further verify this observation, we deleted *AGE3* in a constitutively active calcineurin signaling mutant (DSY2146) that is resistant to FCZ even in the absence of extracellular CaCl_2_ [19]. Deleting *AGE3* in this constitutively active calcineurin mutant restored FCZ sensitivity both in the absence and in the presence of extracellular CaCl_2_ (Figure 2B). These results suggest that constitutive calcineurin signaling does not rescue the *age3*-dependent FCZ sensitivity. The results also support an argument that *AGE3* and calcineurin-dependent processes could be linked (see discussion).

### Pharmacological compromise of ARF cycling converts FCZ into a fungicidal drug in *C. albicans*

**Figure 3:**
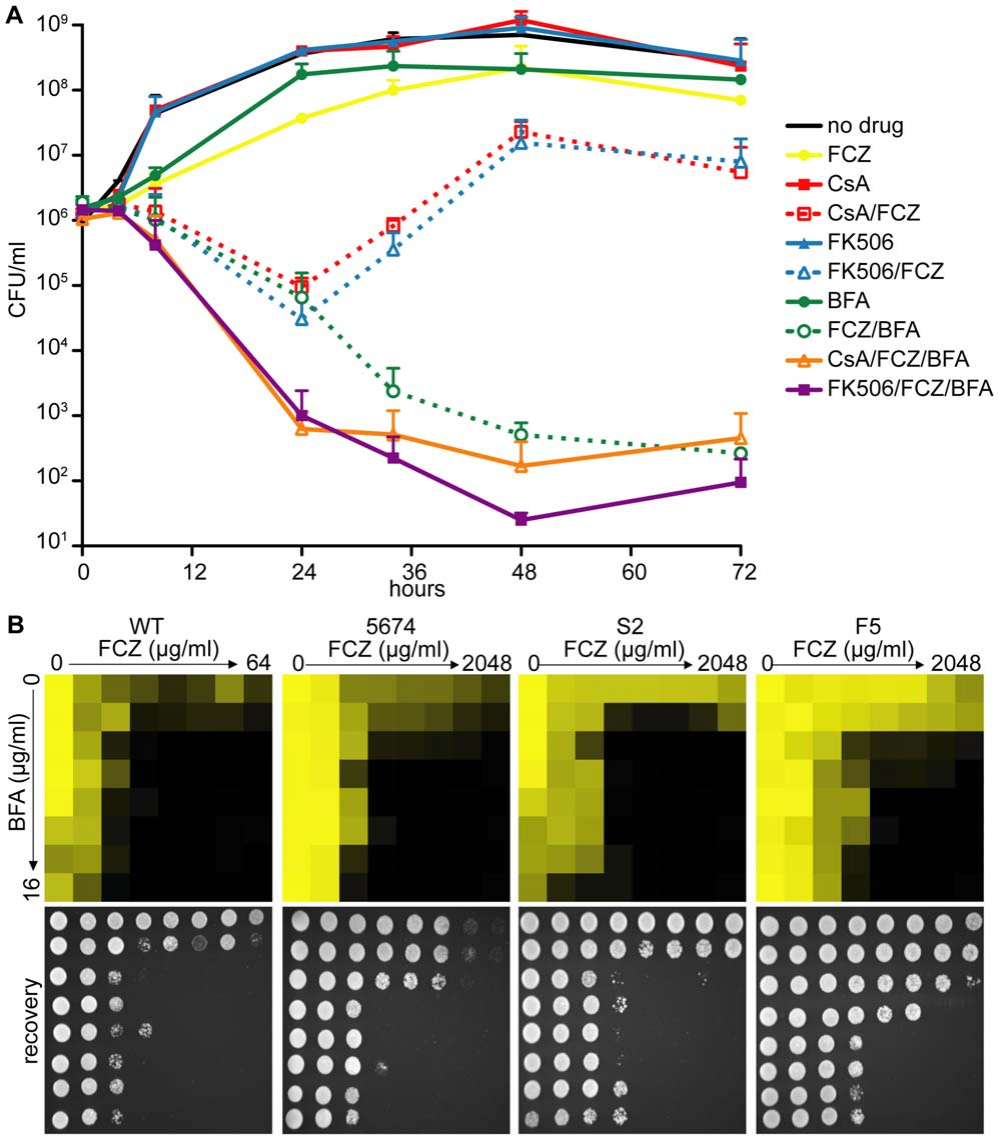
Pharmacological inhibition of ARF cycling results in a potent, fungicidal synergy in combination with FCZ in *C. albicans*. (A) Time-kill curves demonstrating that, while combining FCZ and BFA was initially equally efficacious in pathogen killing compared to combining FCZ and calcineurin inhibitors (FK506 or CsA), extended drug exposure only remained efficacious in pairwise BFA/FCZ combinations. Triple drug combinations of BFA/FCZ/calcineurin inhibitors were only initially (24 hours) more efficacious, but at 72 hours appeared equally efficacious compared to BFA/FCZ. The assay was done in YPD media. Drugs were used at 10 µg/ml for FCZ, 15 µg/ml for BFA, 1 µg/ml for CsA and 1 µg/ml for FK506. (B) Dose-matrix titration assay confirming the FCZ/BFA synergy in WT and drug resistant clinical isolates 5674, S2 and F5 in rich YPD media (top). Dose-matrix titration plates were incubated for 72 hours after which aliquots of each well were spotted on fresh YPD recovery plates (bottom). No-growth of recovery plates confirmed fungal cell death of the drug synergy. Recovery plates were incubated for 24 hours. Dose-matrix titration assays were analyzed as described for MIC assays in Figure 1A.

Given *AGE3*'s role in sensitivity to various drugs and its implication in clinically relevant processes such as drug resistance, targeting either *AGE3* or its biological process seemed a plausible avenue for combination therapies to render FCZ fungicidal. The *S. cerevisiae* homolog of *C. albicans AGE3* is *GCS1*, which encodes an ARF GAP (ADP-Ribosylation Factor GTPase Activating Protein) [37]. ARFs are small G-proteins of the Ras GTPase superfamily that cycle between an active GTP-bound and an inactive GDP-bound state. ARF guanine nucleotide cycling, and hence function, is regulated by GAPs and GEFs (guanine nucleotide exchange factors) [38]. ARFs are involved in a variety of processes including vesicle trafficking (Golgi-to-ER retrograde vesicle trafficking, trans Golgi network-endosomal transport, transport from the Golgi to the membrane) and actin cytoskeleton organization [39],[40],[41],[42]. Brefeldin A (BFA), a metabolite from the fungus *Penicillium decumbens*, is a noncompetitive inhibitor of ARF activity. Protein crystal structures showed that BFA binds to a ternary complex of ARF-GDP-GEF, thus stabilizing this otherwise transient protein-protein interaction [43],[44],[45],[46],[47],[48]. Given that ARF cycling is a well-established target of BFA, we reasoned that BFA might be an ideal drug to synergize with FCZ in WT *C. albicans* by chemically mimicking the *age3*-dependent FCZ sensitivity. As illustrated by time-kill curves in Figure 3A, combining FCZ with BFA resulted in a potent fungicidal synergy in WT *C. albicans*, while either drug alone had only minor effects on cell growth. To compare the BFA/FCZ synergy to a well-established fungicidal synergy, we repeated the time-kill curves with CsA in combination with FCZ. CsA/FCZ co-treatment resulted in a similarly strong synthetic phenotype after 24 hours. This observation corroborates previous findings that calcineurin inhibition plus FCZ results in a potent fungicidal combination as assayed at 24 hours of drug treatment [19]. A triple drug combination of FCZ/BFA/CsA was significantly more efficacious than either FCZ/CsA or FCZ/BFA alone at 24 hours. However, when monitored for more than 24 hours, conditions that have not previously been reported [19], cells treated with FCZ/CsA could recover and resume growth, while cells treated with FCZ/BFA or FCZ/BFA/CsA could not resume growth above the detection limit (≈10 cells/ml). At 72 hours, drug combinations of either FCZ/BFA or FCZ/BFA/CsA appeared equally efficacious with no evidence of growth, while cells treated with FCZ/CsA continued to proliferate. Similar results were obtained when another calcineurin inhibitor, FK506, was combined with FCZ. These results suggest that while the combination of calcineurin inhibitors and an azole is initially efficient in pathogen killing, over prolonged drug treatment, combining ARF inhibitors with azoles is more efficacious.

A dose-matrix titration assay measuring growth of treated cells confirmed the synergy between FCZ/BFA (Figure 3B, Table S3). We also tested whether the FCZ/BFA synergy is still effective in *C. albicans* drug resistant clinical isolates and found that, although considerably higher concentrations of FCZ were needed, there was synergy in isolates F5 and S2 as well as in isolate 5674, which carries a gain-of-function mutation in *TAC1*, a transcriptional activator of *CDR* drug efflux pump genes [49]. Thus, genetic compromise of ARF cycling by deleting *AGE3* abrogated FCZ tolerance, while pharmacological compromise of ARF cycling by adding BFA converted the fungistatic drug FCZ into a fungicidal agent in WT and FCZ-resistant *C. albicans* clinical isolates. Together, this suggests that the process of ARF cycling becomes essential during cell membrane stress in this pathogen.

### Combining ARF inhibition with other drugs across fungal species

**Figure 4:**
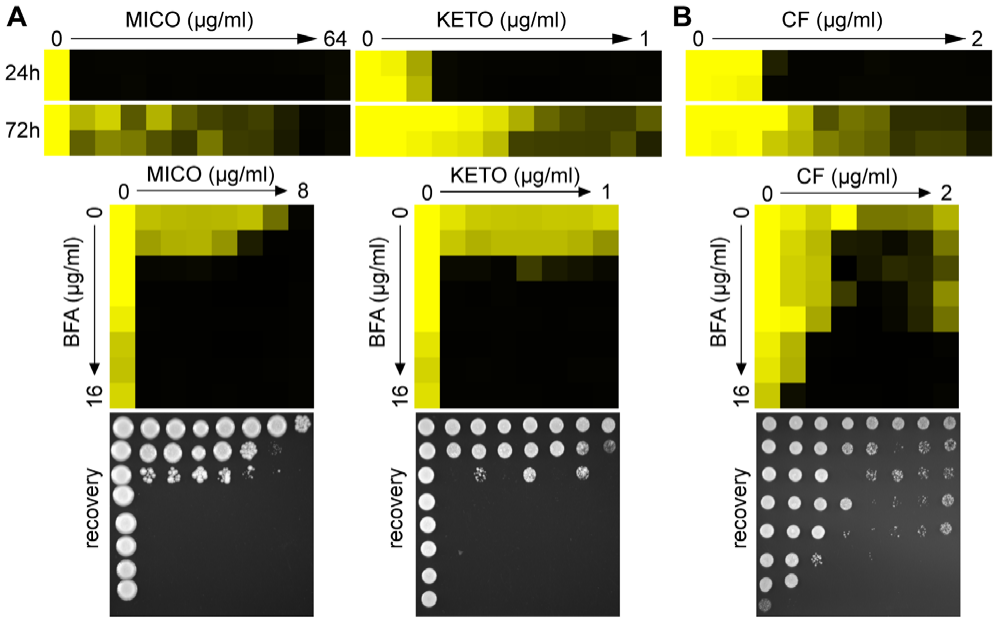
Pharmacological compromise of ARF cycling synergizes with various azoles as well as the cell wall inhibitor CF. (A) MIC assays in YPD media demonstrated that WT *C. albicans* shows tolerance to MICO and KETO (top, compare 24 hours to 72 hours MIC readings). Combining BFA with either MICO or KETO resulted in a similarly potent fungicidal combination compared to BFA/FCZ (bottom). (B) CF showed trailing growth in rich YPD media (top) and synergized with BFA in a dose-matrix titration assay (bottom) against WT *C. albicans*. MIC and dose-matrix titration assays were performed and analyzed as described in Figure 1A and 3B, respectively.

Importantly, genetic or pharmacological compromise of ARF cycling did not appear to significantly affect the cells' initial response to FCZ (Figure 1A, 3B, Table S2). Instead, ARF cycling inhibition seems to act on tolerance. To test whether the effect of BFA on tolerance is observed in combination with other azole drugs, we tested miconazole (MICO) and ketoconazole (KETO) in combination with BFA, because MICO and KETO alone did show a tolerance effect (Figure 4A). When tested in a dose-matrix titration format, BFA synergized with MICO and KETO against WT *C. albicans*, independently of which media were used (Figure 4A, Figure S2).

Although CF is generally considered fungicidal in *C. albicans*, we tested whether BFA would synergize with CF. Recent reports showed that *C. albicans* can start to grow at supra MIC concentrations of CF, an outcome referred to as the paradoxical or trailing growth effect [50],[51]. BFA did synergize with CF against WT *C. albicans* in a dose matrix titration assay at supra MIC concentrations of CF (Figure 4B). Together, these results indicate that genetic and pharmacological compromise of ARF cycling influences not only cell membrane stress, but also cell wall stress.

**Figure 5:**
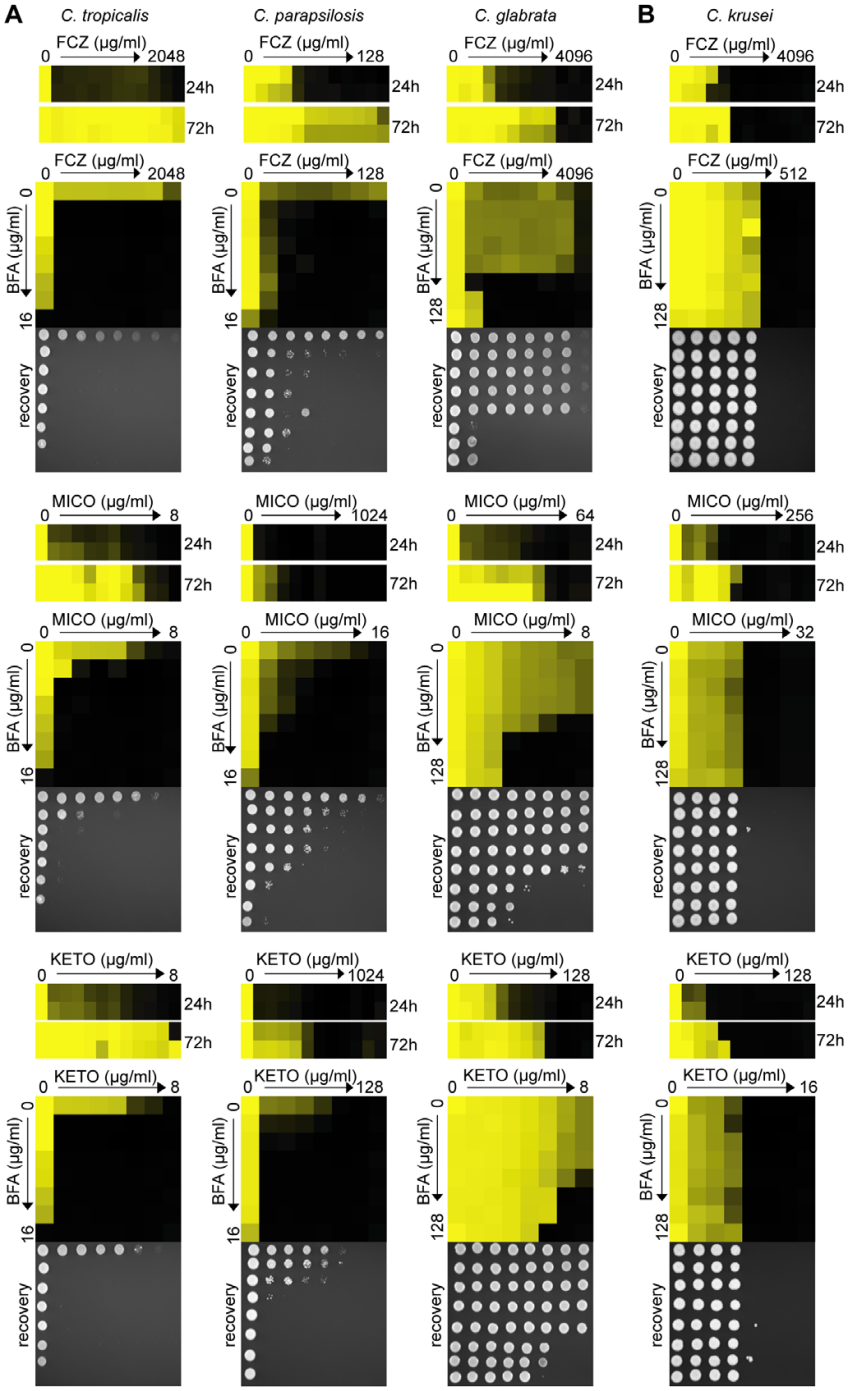
BFA synergizes with different azoles in pathogenic non-*albicans Candida* species. (A) When treated with FCZ, MICO or KETO, *C. tropicalis*, *C. parapsilosis* and *C. glabrata* isolates showed prominent growth above the initial MIC reading after extended incubation (24 hours vs. 72 hours). Dose-matrix titration assays confirming that BFA synergized with the three azoles in all three species. (B) No obvious tolerance effect was observed in *C. krusei* to any azoles tested and no synergy was observed when BFA was combined with these azoles. MIC and dose-matrix titration assays were performed and analyzed as described in Figure 1A and 3B.

To examine whether BFA's inhibition of azole tolerance is conserved across other pathogenic *Candida* species, we tested BFA/drug interactions in *C. tropicalis*, *C. parapsilosis*, *C. glabrata* and *C. krusei*. Together, these species account for ∼30–40% of all *Candida* isolates causing invasive infections; furthermore, *C. glabrata* and *C. krusei* are notoriously difficult to treat with FCZ [7],[8],[12]. *C. tropicalis*, *C. parapsilosis* and *C. glabrata* showed tolerance in the presence of FCZ, MICO and KETO (Figure 5A); these azoles synergized with BFA in dose-matrix titration assays with these fungi. FCZ also synergized with BFA in the non-pathogenic yeast *S. cerevisiae* (data not shown). On the other hand, *C. krusei* did not show an obvious tolerance effect (i.e. >2 fold variations between 24 hours and 72 hours MIC readings), which could explain why BFA did not synergize with azoles in this pathogen (Figure 5B).

**Figure 6:**
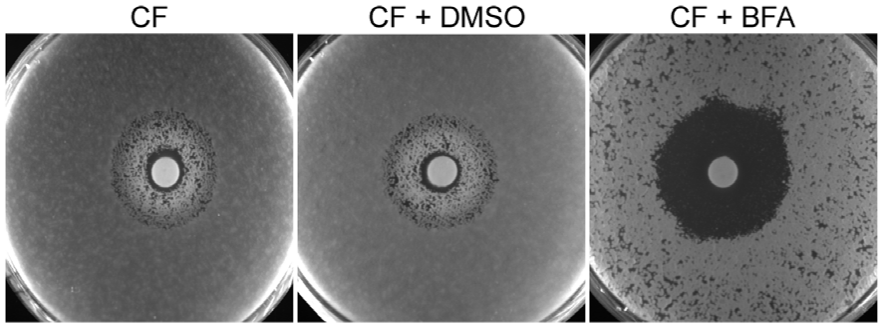
BFA synergism with the fungistatic cell wall inhibitor CF in *A. fumigatus*. BFA/CF interaction in an *A. fumigatus* disk diffusion assay on half-strength YPD media. CF alone creates an inhibition zone that still allows fungal growth. Combining CF and BFA abrogated growth within that zone. Discs containing 160 µg CF were applied after 10^5^ conidia were plated on plates containing water only, vehicle control (DMSO) or BFA (16 µg/ml), as indicated. Plates were incubated for 48 hours.

To further investigate ARF cycling inhibition as a mechanism of abrogating drug tolerance in non-*Candida* human fungal pathogens, we asked whether BFA would synergize with FCZ or CF against *A. fumigatus*. Consistent with the idea that ARF cycling inhibition acts primarily on tolerance, a disc diffusion assay in *A. fumigatus* demonstrated that BFA synergized with CF, a drug that is generally fungistatic in *A. fumigatus* (Figure 6). CF treatment created an inhibition zone against *A. fumigatus* where cells could still grow within that zone due to the fungistatic nature of CF. In contrast, when BFA was combined with CF, not only was the size of that inhibition zone increased, but growth within that zone was also remarkably reduced. A MIC assay in defined synthetic media independently confirmed that CF synergized with BFA against *A. fumigatus* (Table S3). On the other hand, we found that BFA did not synergize with FCZ, a drug that is considered fungicidal in *A. fumigatus* (data not shown) [12]. In general, pharmacological compromise of ARF cycling in *A. fumigatus* was not as efficacious as in *Candida* species, possibly because BFA on its own had a more pronounced impact on growth of *A. fumigatus* compared to *Candida* species (Figure 6, Table S3). Taken together, these results show that ARF cycling inhibition can couple with different fungistatic drugs in many pathogenic fungi to generate potentially fungicidal activity.

### Core transcriptional responses to FCZ and CF treatment are not significantly affected in the absence of *AGE3*

Analysis of transcriptional regulation has frequently been used to elucidate which cellular processes are linked to drug sensitivity or drug resistance in *C. albicans* [35],[52],[53]. We therefore performed microarray studies to test how transcriptional regulation is altered in *age3* mutants. We first compared WT to *age3* cells in the absence of drugs and found that 23 genes were differentially regulated when *AGE3* was absent (Table S4). Among those 23 genes were five GPI-anchored cell wall proteins (*ECM331*, *PGA13*, *CRH11*, *SAP9*, *PGA26*) and two genes with phospholipase activity (*FGR22* and *PI-PLC*). Because none of these genes have been linked to FCZ tolerance, it is currently unclear how they might influence the *age3*-dependent drug phenotypes.

We next analyzed the transcriptional response to FCZ. A typical transcriptional signature to FCZ is upregulation of ergosterol genes, presumably to compensate for depletion of these membrane lipids [54],[55]. This core response to FCZ was not changed in the absence of *AGE3*. All ergosterol genes that became significantly upregulated in FCZ-treated WT cells were similarly upregulated in FCZ-treated *age3* cells (Table S5). Besides this core response to FCZ, clustering analysis of genes that were significantly regulated (>2 fold expression change and *p*-value <0.05) further confirmed that the overall transcriptional response to FCZ was very similar in WT and *age3* cells (Figure S3A, Table S6).

A similar lack of *AGE3*-dependent transcriptional consequences could be observed in microarray experiments when the cell wall inhibitor CF was used. Clustering analysis revealed that CF-treated WT cells and CF-treated *age3* cells showed an almost identical transcriptional response, with a significant overlap (*p*-value 4.8×10^−194^) of differentially regulated genes (Figure S3B, Tables S7, S8). This overlap of 168 genes showed further statistically significant similarity (*p*-value 1.8×10^−7^) when compared to core *C. albicans* CF-responsive genes previously identified in two independent studies [52],[56]. Therefore, the core transcriptional response to CF in *C. albicans* seems not to depend on *AGE3*. In summary, these microarray experiments suggest that deleting *AGE3* does not cause major transcriptional changes in the presence of two different drug classes and further indicates that post-transcriptional processes might play a more dominant role in terms of ARF cycling-dependent drug phenotypes.

### The *age3* mutant is avirulent in WT mice

**Figure 7:**
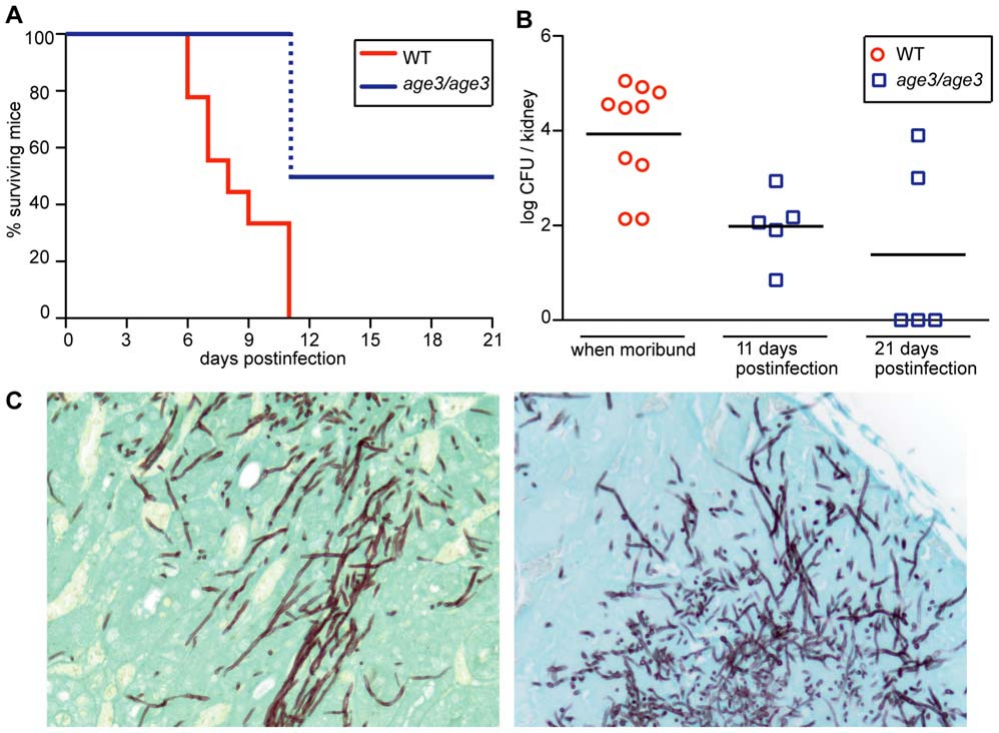
Genetic compromise of ARF cycling in *C. albicans* results in avirulence in a WT mouse model of disseminated disease. (A) *C. albicans* WT-infected mice become gradually moribund up to day 11, while mice infected with *age3* mutants did not show any clinical signs until the end of the experiment on day 21. The dotted blue line indicates that half of the *age3* mutant-infected mice were sacrificed to compare fungal load. Those mice were not moribund. (B) On average, fungal load of WT-infected mice, when moribund, is significantly higher compared to mutant fungal burden taken at indicated times. (C) Kidney section of WT-infected mice (left) showing fungal hyphal formation, which is also seen in mutant-infected kidneys (right). Kidneys were collected on day 11 for histological examination. Ten mice were used per experimental group and monitored according to approved standards.

To evaluate whether *AGE3* is a good drug target *in vivo*, we first injected *age3* mutant cells into WT B6 mice, and found that *age3* cells are avirulent in this mouse model. While 100% of mice infected with WT *C. albicans* became moribund within 11 days post-infection, none of the *age3* mutant strain infected mice became moribund during the same time frame (Figure 7A). To test whether mice infected with *age3* cells had cleared the infection, we sacrificed half of the mutant group on day 11 to analyze kidney fungal burden (Figure 7B). On average, kidney fungal load was significantly reduced (*p*-value<0.01, Mann Whitney test) in the *age3* group compared to moribund WT-infected mice. We continued with the other half of *age3* mutant-infected mice until the end of the experiment (day 21), but again found that none of the *age3*-infected mice became moribund. Comparing fungal load from these mice showed that three mice had cleared the infection, while two mice had a fungal load that was comparable to WT-infected mice. On average, however, there were significantly fewer *age3* cells recovered from the host compared to moribund mice infected with WT *C. albicans* (*p*-value <0.02, Figure 7B).

One key virulence factor in *C. albicans* pathogenicity is hyphal formation [57]. Because Arnold Bito and coworkers (Lettner T., Zeidler U., Gimona M., Breitenbach M., Bito A., manuscript submitted, personal communication from A. Bito) have observed some hyphal formation defects on solid media as well as defects in invasive growth in *age3* mutants, we collected kidneys for histological examination on day 11 from both WT-infected and *age3*-infected mice. No obvious difference was found between kidney sections recovered from WT or from *age3*-infected mice (Figure 7C). In all kidney sections examined, *age3* cells could be observed as elongated hyphal structures. Therefore, it remains unclear why *age3*-infected mice with a high fungal burden did not show any clinical signs, but might indicate that additional virulence factors are affected in *age3* mutants.

### Genetic inhibition of ARF cycling results in attenuated virulence in an immunocompromised mouse model of disseminated candidiasis and FCZ treatment is significantly more efficacious when ARF activity is genetically compromised

**Figure 8:**
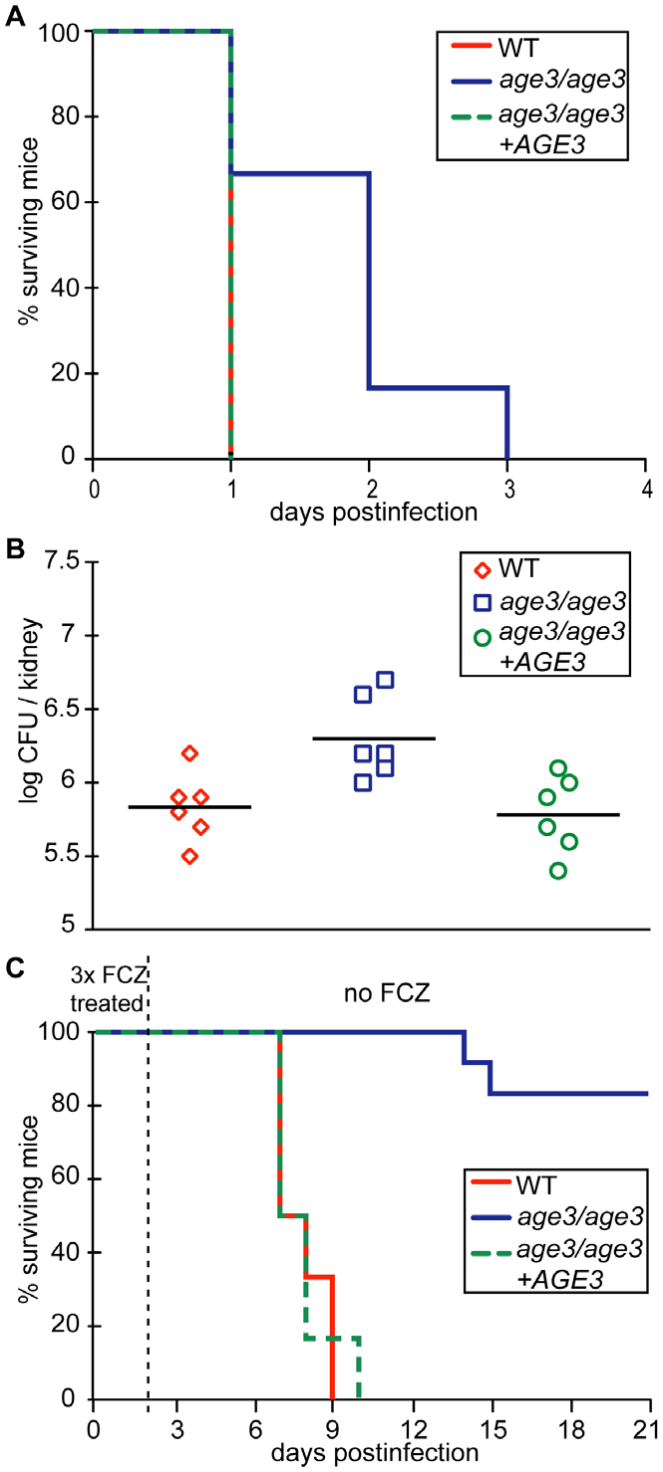
*age3* mutants are attenuated in virulence in A/J mice and FCZ treatment significantly extends survival of *age3*-infected A/J mice. (A) *age3* mutant-infected mice survive significantly longer with a median survival of two days versus one day for WT and revertant-infected mice. Six mice were used per experimental group. (B) Fungal kidney burden was examined from moribund mice and was significantly higher in *age3* mutant recovered cells compared to WT or revertant control groups. (C) A short FCZ therapy (4.5 mg/kg intraperitoneally once immediately after fungal infection, once on day one and once on day two post fungal infection) is significantly more efficacious when ARF cycling is genetically compromised as only the majority (83%) of mutant-infected mice survive until the end of the experiment (day 21). Six mice were used for WT and revertant groups and 12 mice for the *age3* mutant group.

To help evaluate the therapeutic potential of FCZ treatment of *age3* mutant-infected mice, we required a mouse model where *age3* cells retain at least partial virulence. Cells of the *C. albicans* strains WT, *age3* mutant and *age3* revertant were therefore injected in an immunocompromised C5-deficient A/J mouse model [58]. In this very sensitive animal system, *C. albicans* WT and revertant-infected A/J mice became rapidly moribund after 20 to 24 hours, while *age3* mutant-infected A/J mice survived significantly longer, with a median survival of two days (*p*-value<0.02, Log-rank test) (Figure 8A). However, comparing fungal burden indicated that mice infected with *C. albicans age3* cells accumulated a significantly higher fungal load when moribund (*p*-value <0.02), with hyphal formation still observed in the *age3* mutant-infected mice (Figure 8B, data not shown).

To test whether genetic compromise of ARF activity holds therapeutic potential, we repeated this experiment and injected WT, *age3* and the revertant strains in A/J mice to compare survival lengths after two days of FCZ therapy. FCZ administration extended survival of all three groups (*p*-value <0.001), but was significantly more efficacious in the mutant-infected mice, extending median survival time more than 10-fold compared to 7.5-fold median survival time extension for both the WT and revertant groups (*p*-value <0.01 for posthoc comparison between *age3* mutant group with and without FCZ, versus *p*-value >0.05 posthoc comparison between WT with and without FCZ or *p*-value >0.05 for posthoc comparison between revertant with and without FCZ, Dunnett's Multiple Comparison) (Figure 8C). In summary, we conclude from these *in vivo* experiments that genetic compromise of ARF cycling results in avirulence in WT mice, significant attenuation in virulence in an immunocompromised mouse model and further suggests that FCZ treatment in A/J mice is more efficacious when ARF cycling is genetically compromised.

## Discussion

Identifying new drug targets is an important step in the challenging task of developing new antifungal therapies, which are urgently needed due to the emergence of drug resistance to every class of antifungals currently in clinical use [11],[59]. Using a comprehensive reverse-genetics screen, we identified Age3p and the process of ARF cycling as potential new drug targets. We further established a widely conserved, potent fungicidal chemical synergy between the ARF cycling inhibitor BFA and several fungistatic drugs, and, through two murine infection models, validated the potential of ARF cycling as an antifungal target.

To render FCZ fungicidal through combination with other drugs, we applied a large-scale chemical-genomic approach in *S. cerevisiae* and found 22 FCZ-cidal genes (Table S1) [29]. The *S. cerevisiae* screen proved to have predictive power in that 12 of the 22 genes (57%) identified in *S. cerevisiae* were validated in *C. albicans* with an increased FCZ sensitivity (Figure S1, Table S2). Among those genes, only *CDR1* and *ADA2* have previously been linked to FCZ sensitivity in *C. albicans* [52],[60]. On the other hand, our screen linked four genes to FCZ resistance in the pathogen (*NUP84*, *ERG11*, *GCN5* and *NGG1*). One of those genes, *ERG11*, has previously been linked to FCZ resistance in *C. albicans* [61]. Of note, Sanglard and coworkers have shown that the *C. albicans erg11* mutant was sensitive to a variety of drugs, including BFA [61]. Two other *C. albicans* genes that have been linked to FCZ resistance in our screen (*GCN5* and *NGG1*) are part of the SAGA, a conserved transcriptional co-activator [62]. Our data therefore expand the known events of transcriptional rewiring between *S. cerevisiae* and *C. albicans* in regards of drug resistance [63],[64], as another subunit of the SAGA complex, Ada2p, has been linked to increased drug sensitivity in our screen as well as in two previous studies [52],[60]. The observation that different subunits of the same transcriptional co-activator complex regulate the opposing phenotypes of drug sensitivity or drug resistance illustrates the extreme adaptability and flexibility of transcriptional regulatory networks in fungi.

Given that diverse essential processes exhibit significantly different regulation in *S. cerevisiae* and *C. albicans* [65],[66],[67], reorganization of transcriptional regulation might in general account for *C. albicans* mutants that showed different drug phenotypes compared to the yeast prediction. Despite these considerations, budding yeast genetics remains a convenient and powerful approach to predict phenotypes in pathogenic fungal species, at least until similar sophisticated screening techniques become available in the pathogen itself.

An alternative speculation why the success rate of our screen was not higher is that the gene encoding *ERG11*, the target of FCZ in both species, is essential only in *S. cerevisiae* [29],[61]. Thus, there may be redundant mechanisms for sterol biosynthesis in *C. albicans*, a suggestion that is further supported by the observation that another ergosterol biosynthesis gene, *ERG24*, is essential in *S. cerevisiae*, but not in *C. albicans* [68].

Chemical compromise of ARF cycling appeared far more potent than genetic compromise. When *age3* mutants were treated with FCZ, the number of viable cells was reduced in time-kill curve experiments by less than one log value, whereas combining FCZ with BFA reduced the number of viable WT cells more than one log value over the same time (Figure 1C, Figure 3A). Similarly, chemical compromise appeared more potent than genetic compromise in terms of synergy with CF. The *age3* mutant was only 2-fold more sensitive to CF than WT. On the other hand, combining BFA with CF resulted in a 32-fold reduction in sensitivity to CF in WT cells (Figure 1D and 1E, Figure 4B). These differences likely reflect that, while chemical interference with ARF function inhibits potentially all ARF GEFs, genetic inactivation was restricted to one ARF GAP (*AGE3*). Thus, it remains possible that some aspects of ARF signaling continue to function in the absence of *AGE3* under drug conditions, a suggestion that is supported by work in yeast that demonstrated that several ARF GAPs provide redundant functions [39],[40].

The finding that both genetic and pharmacological blockage of ARF function result in increased azole sensitivity suggests that, among the multiple cellular roles described for the yeast homologue of *AGE3*, defects in proper ARF cycling and, therefore, defects in intracellular vesicle trafficking are responsible for drug phenotypes. One hypothesis to explain how incorrect vesicle trafficking could result in *age3*-dependent drug sensitivity is mislocalization of drug pumps. However, *CDR1* and *MDR1*, two major drug pumps in *C. albicans* appear not be involved, as Arnold Bito and coworkers (Lettner T., Zeidler U., Gimona M., Breitenbach M., Bito A., manuscript submitted, personal communication from A. Bito) observed that *CDR1* and *MDR1* pumps are correctly localized to the plasma membrane in *age3* mutants. They further established that *CDR1* drug pump activity was not affected in the absence of *AGE3*.

Another plausible explanation that could account for the azole sensitivity of *age3* mutants is defects in the biosynthesis of ergosterol or problems in transporting this membrane lipid to the cell membrane. Our microarray experiments provided evidence that the target pathway of azoles is not affected transcriptionally in the presence or the absence of FCZ (Figure S3A, Tables S5 and S6). We further found that the amount of ergosterol is similar in the plasma membrane of *age3* mutants compared to WT (our unpublished data). These findings together with epistasis experiments showing that deletion of *AGE3* restored azole sensitivity in different clinical isolates, suggest that the azole sensitivity of *age3* cells is unlikely to depend on established mechanisms.

Finally, in support of the vesicle transport hypothesis is the observation that while *age3* cells showed slightly increased sensitivity to different cell wall perturbing agents (CF and calcofluor white), the most impressive effect besides azoles was observed when *age3* cells were treated with hygromycin B (Figure 1D). Hygromycin B sensitivity is usually linked to glycosylation defects [31],[32]. Therefore, it remains possible that some glycosylated proteins, including GPI-anchored proteins that normally reside in the cell wall, are not properly localized in *age3* cells. How precisely defects in vesicle trafficking influence the observed drug phenotypes and whether drug sensitivity is caused by a general aspect of the secretory pathway or of a particular cell membrane or wall protein remains to be determined.

Drug resistance and virulence are two important biological aspects of pathogenic fungal species. While different fungal drug resistance mechanisms are now well understood [69],[70], various virulence-related attributes have been described that help *Candida* to cause infections [57],[59],[71]. Whereas genes critically involved either in drug resistance or virulence are attractive drug targets [59], an undeniably better option is targeting genes that are involved in both processes. The broad-range sensitivity to azoles and an echinocandin together with *in vivo* data showing that *age3* mutants are avirulent in WT and exhibit significantly attenuated virulence even in an immunocompromised mouse model, indicates that Age3p and the process of ARF cycling is one such option. We further explored the therapeutic potential of ARF cycling inhibition by demonstrating that, in A/J mice, FCZ treatment was significantly more efficacious when ARF activity was genetically compromised.

One of our major problems was to reproduce the potent *in vitro* synergy of FCZ/BFA in animal models as we observed that a FCZ/BFA combination failed to rescue A/J mice infected with WT *C. albicans* (data not shown). One reason why the *in vitro* synergy failed to translate to *in vivo* conditions could be that BFA has low bioavailability characteristics [72] and efforts to chemically improve these unfavorable properties have not been successful so far [73],[74]. The ability of BFA to induce apoptosis in cancer cells has stimulated an interest for developing BFA as an anti-cancer therapeutic agent [75],[76],[77],[78],[79],[80]. Increasing evidence also shows that a variety of small G protein signaling pathways of the Ras superfamily, like RHO, RAS and ARF, have been linked to tumorigenesis [81],[82],[83]. Thus, despite being evolutionarily conserved, targeting ARF activities could be beneficial not only for antifungal, but also for cancer therapies. Clearly, one future challenge lies in finding fungal-specific ARF activity inhibitors that retain favorable bioavailability *in vivo*, a challenge that can now be approached cost- and time-effectively through virtual screening with both mammalian and yeast crystal structures of different ARF cycling proteins at hand [43],[44],[45],[46],[47],[48],[84],[85].

The *age3* mutant lost tolerance to FCZ (Figure 1). This phenomenon of losing tolerance under membrane stress is not unique to ARF cycling. Previous work established that when HSP90 or calcineurin is genetically or pharmacologically inhibited in different fungal species, cells show a similar loss of tolerance and are not able to survive membrane stress [17],[18],[22],[86]. Additional phenotypes that calcineurin inhibition shares with ARF inhibition include synergism with azoles and echinocandins as well as reduced virulence in the bloodstream of the host [17],[19],[87]. These overlapping phenotypes, together with our epistasis experiments that showed that deleting *AGE3* overrides constitutive calcineurin signaling, provide some arguments that ARF cycling and HSP90/calcineurin-dependent processes could be coupled. Although overlapping functions cannot be excluded, several lines of evidence support the current model that ARF and HSP90/calcineurin inhibition constitute two distinct mechanisms contributing to drug tolerance. First, a triple combination of azole/calcineurin/ARF inhibition is more efficacious than either pairwise combination at 24 hours. Second, consistent with previous observations that long term azole resistance evolves towards HSP90/calcineurin-independency [20], cells treated with calcineurin inhibitors plus FCZ recover after prolonged incubation, while BFA/FCZ treated cells do not. Finally, if ARF cycling is in fact coupled to HSP90/calcineurin signaling, then we might expect *age3* mutants to copy other HSP90/calcineurin phenotypes besides the demonstrated reduced virulence and drug sensitivity. One explanation, for instance, why calcineurin mutants are avirulent is that they do not survive in the presence of FBS or Ca^2+^ ions present in host serum [19],[88]. We found, however, that *age3* mutants can survive FBS and Ca^2+^ stresses (data not shown).

More work is therefore required to elucidate how ARF G protein signaling relates to HSP90/calcineurin and other signaling pathways that share ARF-related phenotypes. With four ARF proteins, six ARF GAPs and seven ARF GEFs identified in yeast so far [89], this provides a rich resource for further investigations into which aspects of vesicle transport and ARF G protein signaling are responsible for two important aspects of the biology of pathogenic fungal species.

## Materials and Methods

### Strains, plasmids, primers and culture conditions

All strains, primers and plasmids used in this study are described in supplementary Table S9, S10 and S11, respectively. *C. albicans* mutants were constructed either with the UAU1-transposon insertion strategy [90] or by deleting the coding sequence of genes (Table S9). For insertion and deletion mutant construction, at least two mutants independently derived from two distinct heterozygous mutants were analyzed in each case. With regards to the *AEG3* gene (*ORF19.3683*), we propose to use *AGE3* as standard name for the *C. albicans* homolog of *S. cerevisiae's GCS1*, because another gene (*ORF19.5059*) has already been named ‘*GCS1*’ in the Candida literature [91]. For the *AGE3* deletion mutant, long 100-mer primers flanking up- and downstream sequences, respectively, of the coding sequence of *AGE3* were used to amplify marker cassettes pFA-*HIS1* and pFA-*ARG4* [92]. Transformation was carried out according to standard protocols [93] and selected on synthetic media (2% dextrose, 6.7% yeast nitrogen base without amino acids, 2% agar) containing the necessary auxotrophic supplements. Correct marker integration was PCR-verified as described [94]. As genetic manipulation in *C. albicans* can frequently lead to aneuploidy [95],[96], we verified the absence of any chromosomal rearrangements by Comparative Genome Hybridization (CGH) and found that deletion mutants of *AGE3* were aneuploidy free (Figure S4). For constructing the *AGE3*-revertant strain, the SAT1 flipper cassette was used [97]. Briefly, the *AGE3* coding sequence including upstream and a short downstream flanking sequence was amplified with primers oEE242/oEE243, *Kpn*I/*Xho*I-digested and cloned into the *Kpn*I/*Xho*I-digested pSFS2A plasmid, resulting in plasmid pCaEE25, which was sequenced. A long downstream flanking sequence of *AGE3* was amplified with primers oEE237/oEE238, *Not*I/*Sac*II-digested and then cloned into the *Not*I/*Sac*II-digested plasmid pCaEE25, resulting in plasmid pCaEE27. Following *Kpn*I/*Sac*II double digestion of plasmid pCaEE27, this digestion was transformed directly into the *age3* deletion strain. Selection was done on YPD plates containing 200 µg/ml nourseothricin, as described [67] and PCR-verified. Counterselection of the nourseothricin marker was done as described [67].

The nourseothricin marker cassette [97] was also used to delete *AGE3* in FCZ-resistant strains F5, S2 and DSY2146. Briefly, a long upstream coding sequence of *AGE3* was first amplified from pEE27 with primers oEE235/oEE236, *Kpn*1/*Xho*1 digested and then cloned into the *Kpn*1/*Xho*1 digested plasmid pEE27, therefore replacing the coding sequence of *AGE3*. The resulting plasmid was designated pEE43.

*Candida* strains were routinely cultured at 30°C in either rich YPD media (1% yeast extract, 2% peptone, 2% dextrose, supplemented with 50 µg/ml uridine) or RPMI-MOPS media (RPMI-1640, SIGMA, supplemented with 0.3 g/l L-glutamine, 50 mg/ml uridine, 2% glucose, pH adjusted with MOPS buffer to 7.0). Media plates were supplemented with 2% agar. *A. fumigatus* was cultured in half-strength YPD media (not enriched with uridine) or RPMI-MOPS media (not enriched with uridine). Media plates were supplemented with 1.5% agar.

### Antifungal susceptibility testing

Because drug susceptibility results did not differ significantly between WT *C. albicans* strains SC5314 (isolate) and SN95 (a standard laboratory auxotrophic mutant used to construct deletion mutants) [98] (Table S2), WT usually refers to SN95 unless indicated otherwise. Drug stock solutions were prepared using ethanol/10% Tween 20 as solvent for FK506 (5 mg/ml), cyclosporin A (25 mg/ml), DMSO for fluconazole (300 mg/ml), miconazole (100 mg/ml), ketoconazole (16.6 mg/ml), itraconazole (10 mg/ml), amphotericin B (20 mg/ml), calcofluor white (50 mg/ml), brefeldin A (20 mM), hygromycin B (50 mg/ml), and water for caspofungin (10 mg/ml). All drugs were obtained from Sigma, except caspofungin (Merck), fluconazole and itraconazole (both from SpectrumChemical, Mfg Corp, USA). Once in solution, drugs were stored at −20°C. Initial antifungal sensitivity testing with all *C. albicans* FCZ-cidal candidates was done using a modified version of the CLSI (formerly NCCLS) procedure [99]. Briefly, 50 µl of drugs at two-fold the final concentration was serially diluted in flat-bottom 96-well tissue culture plates (Corning Inc., NY, USA) and combined with 50 µl of overnight *Candida* cultures adjusted to 1×10^4^ cells/ml. MIC plates were incubated at 30°C without shaking and optical densities were read at indicated time points with a Tecan Safire plate reader. The MIC was determined by the first well with a growth reduction of >95% in terms of OD_600_ values in the presence of a compound compared to untreated control cells. Before the 72 hours OD_600_ readings, plates were carefully shaken, so that a representative aliquot of 2 µl of each well could be spotted on fresh YPD recovery plates to assess the extent to which cells recover from the drug treatments. Recovery plates were incubated at 30°C between 24 hours and 48 hours before being photographed. Drug susceptibilities of robust hits that resulted from this initial MIC testing were then confirmed on solid media plates containing FCZ, disc diffusion or time-kill curve assays, as described [19].

Dose-matrix titration assays were used to evaluate drug synergies. Briefly, dose-matrix titration assays were done as described for the MIC assays, except that the final volume was 150 µl; 50 µl of three-fold the final drug concentration of drug A was dispensed in two-fold serial dilution steps across seven columns of the plate, 50 µl of three-fold the final drug concentration of drug B was dispensed in two-fold serial dilution steps down seven rows of the plate; 50 µl of overnight *Candida* cultures adjusted to 1.5×10^4^ cells/ml was dispensed in all drug containing wells plus one control well containing no drugs. Synergy of a compound pair was quantified with respect to the Loewe additivity model [100] via the fractional inhibitory concentration index (FIC index) = (MIC_A in combo_/MIC_A alone_) + (MIC_B in combo_/MIC_B alone_), where “MIC_A in combo_” is the MIC of drug A in combination, “MIC_A alone_” is the MIC of drug A alone, “MIC_B in combo_” is the MIC of drug B in combination and “MIC_B alone_” is the MIC of drug B alone, respectively. Compound pairs were classified as synergistic if its FIC index is ≤0.5, the standard threshold [100],[101]. For calculation purposes of the FIC index, MIC values of >1, >2, >8, >16, >64, >128, >2048, >4096 were assumed to be 2, 4, 16, 32, 128, 256, 4096, 8192, respectively. MIC and dose-matrix titration results were visualized with TreeView version 1.6 (http://rana.lbl.gov/EisenSoftware.htm).

*A. fumigatus* disc diffusion assays were done as described [16], with the following modifications. Conidiation was induced on YPD plates incubated at 37°C for seven days. Conidia were then washed off the plates and suspended in PBS+0.1% Tween media before spreading 1×10^5^ conidia on appropriate plates. Discs containing 6.4 µg caspofungin were applied and the plates were incubated at 35°C for 48 hours. *A. fumigatus* MIC assays were done exactly as described [102]. All MIC and dose-matrix titration assays with *Candida* and *Aspergillus* were independently performed on at least two different occasions.

### Virulence studies

Virulence testing of *C. albicans* was done as previously described [58]. Briefly, 8- to 12-week old C57BL/6J or A/J mice (Jackson Laboratories, Bar Harbor, ME) were inoculated via the tail vein with 200 µl of a suspension containing 3×10^5^ *C. albicans* in PBS. Mice were closely monitored over a period of maximally 21 days for clinical signs of disease such as lethargy, ruffled fur, or hunched back. Mice showing extreme lethargy were considered moribund and were euthanized. All experimental procedures involving animals were approved by the Biotechnology Research Institute Animal Care Committee, which operates under the guidelines of the Canadian Council of Animal Care. Statistical analysis of survival curves as well as fungal load was done with GraphPad Prism version 5.0b. For kidney sections, extracted organs were fixed in 10% formaldehyde (Sigma) and processed at the Histology Core Facility at McGill (http://cancercentre.mcgill.ca/) by staining thin slices of tissue sections with Grocot–Gomori methenamine silver to visualize fungal cells.

### Microarray and CGH studies

Comparative Genome Hybridization (CGH) analysis was done as previously described [49] with the following modifications. Genomic DNA was extracted from a *C. albicans* culture grown to saturation with the Qiagen Genomic DNA Extraction kit according to manufacturer's instructions. DNA hybridization was done with the Advalytix SlideBooster for 16 hours at 42°C according to manufacturer's instructions.

For the fluconazole microarray experiment, overnight cultures of *C. albicans* cells were diluted to OD_600_ of 0.05 in fresh YPD media, grown to early logarithmic phase (OD_600_ 0.8) and split into two 50 ml cultures in 250 ml Erlenmayer flasks. One culture was fluconazole treated (600 mg/ml), while the other group received an equal amount of DMSO as control. Cultures were grown for one hour, spun down, the supernatant was removed and the cell pellet was nitrogen-flash frozen and stored at −80°C until further use. RNA was extracted according to the hot-phenol protocol [103]. For the caspofungin microarray experiment, logarithmically growing cells were treated with 125 ng/ml caspofungin for one hour, as previously described [52]. RNA was extracted with the RNase easy kit (Qiagen), as described [67]. Probe labeling, hybridization and slide washing was done as described [104], except that the SlideBooster was used for hybridization. At least three biological replicates including dye swaps were used for each condition on double spotted ORF microarrays (6,394 intragenic 70-mer oligonucleotide probes) [104]. Scanning was done with a ScanArray Lite microarray scanner (Perkin Elmer). QuantArray was used to quantify fluorescence intensities and data analysis was carried out using Genespring v.7.3 (Agilent Technologies). To compare overlap of different gene lists as well as analyzing Gene Ontology enrichment, *p*-values were calculated using the hypergeometric distribution as described in the GO Term Finder Tool web site (http://www.candidagenome.org/cgi-bin/GO/goTermFinder). Gene lists can be found in Tables S4, S5, S6, S7 and S8.

## Supporting Information

Figure S1 Phenotypes of the predicted FCZ-cidal genes in C. *albicans* as determined on rich media containing FCZ. Five-fold serial dilutions starting with an overnight culture diluted to OD_600_ of 0.1 was spotted (2 µl) on YPD or YPD + 2 µg/ml FCZ. Plates were incubated at 30°C for the time indicated.
(5.05 MB TIF)

Figure S2 BFA synergy with different azoles in synthetically defined RPMI media at 30°C. (A) Prior to synergy testing, *C. albicans* WT strain was tested for drug sensitivity, which was measured over time on day one (24 hours), day three (72 hours) and day six (144 hours), respectively. Data was analyzed as in Figure 1A. (B) Optical densities of dose-matrix titration assays were measured on day three (72 hours) or day six (144 hours), respectively. Additionally, spot assays were done on day six. Except for media, assays were performed and analyzed as in Figure 1A and 3B.
(2.15 MB TIF)

Figure S3 Core transcriptional responses to FCZ or CF are not significantly affected in the absence of *AGE3*. (A) Transcriptional analysis under FCZ treatment. Significantly regulated genes (>2 fold change, *p-value *<0.05) were selected when WT was treated with FCZ (WT+FCZ vs. WT) and combined with significantly regulated genes when *age3* cells were FCZ treated (*age3*+FCZ vs. *age3*) to build a cluster tree (top). The same gene list was used to visualize in the Venn diagram (bottom) a significant overlap of core FCZ-responsive genes. (B) Transcriptional analysis under CF treatment. Gene lists were selected in the same way as described for the FCZ treatment to build a cluster tree. The Venn diagram illustrates that there is a significant overlap of core CF responsive genes. Tables S4, S5, S6, S7 and S8 list exact transcript changes for all significantly regulated genes used in the FCZ and CF analysis.
(0.89 MB TIF)

Figure S4 No aneuploidies were detected in the *AGE3* deletion mutant by CGH analysis. Cy3 labeled genomic DNA from either *age3* mutants or strain BWP17 was hybridized to DNA microarrays with Cy5 labeled genomic DNA from the reference strain SC5314. Shown are plots of CGH (comparative genome hybridization) analyses, where the y-axis shows the log2 fluorescence ratio of the mutant strains versus SC5314 and the x-axis shows all chromosomes (1 to R). A single black rhombus represents the log2 fluorescence ratio plotted as a function of its position in the *C. albicans*' assembly 21. In this representation, a 1.5-fold increase in fluorescence ratio (i.e. 3 chromosome copies versus 2) equals a log2 ratio of ∼0.58. (A) CGH shows the known loss of one end of chromosome 5 in strain BWP17. This strain is also auxotroph for *URA3*, *HIS1*, *ARG4*. (B) The prototrophic *age3* mutant does not have any chromosomal rearrangements as determined by CGH.
(7.62 MB TIF)

Table S1 The 22 FCZ-cidal genes in *S. cerevisiae* and the *C. albicans* homologs.
(0.04 MB XLS)

Table S2 Phenotypes of the predicted FCZ-cidal genes in *C. albicans* as determined by MIC assay in YPD media.
(0.04 MB XLS)

Table S3 Drug synergy interaction as determined by FIC index.
(0.04 MB XLS)

Table S4 The genes listed here were significantly regulated (>2 fold, p-value <0.05) in absence of *AGE3*.
(0.04 MB XLS)

Table S5 Fluconazole (FCZ) responsive genes as identified by microarray analysis.
(0.07 MB XLS)

Table S6 This list contains the same genes as Table S5, but the genes are colored here according to the Venn Diagram in Figure S3A (bottom).
(0.07 MB XLS)

Table S7 Caspofungin (CF) responsive genes as identified by microarray analysis.
(0.13 MB XLS)

Table S8 This list contains the same genes as Table S7, but the genes are colored here according to the Venn Diagram in Figure S3B (bottom).
(0.13 MB XLS)

Table S9 Strains used in this study.
(0.04 MB XLS)

Table S10 Primers used in this study.
(0.05 MB XLS)

Table S11 Plasmids used in this study.
(0.03 MB XLS)
