# Supplementary figures and images for: Reverse Genetics in Candida albicans Predicts ARF Cycling Is Essential for Drug Resistance and Virulence
Source: PLoS Pathog. 2010 Feb 5;6(2):e1000753. doi: 10.1371/journal.ppat.1000753 (PMC2816695; doi:10.1371/journal.ppat.1000753)

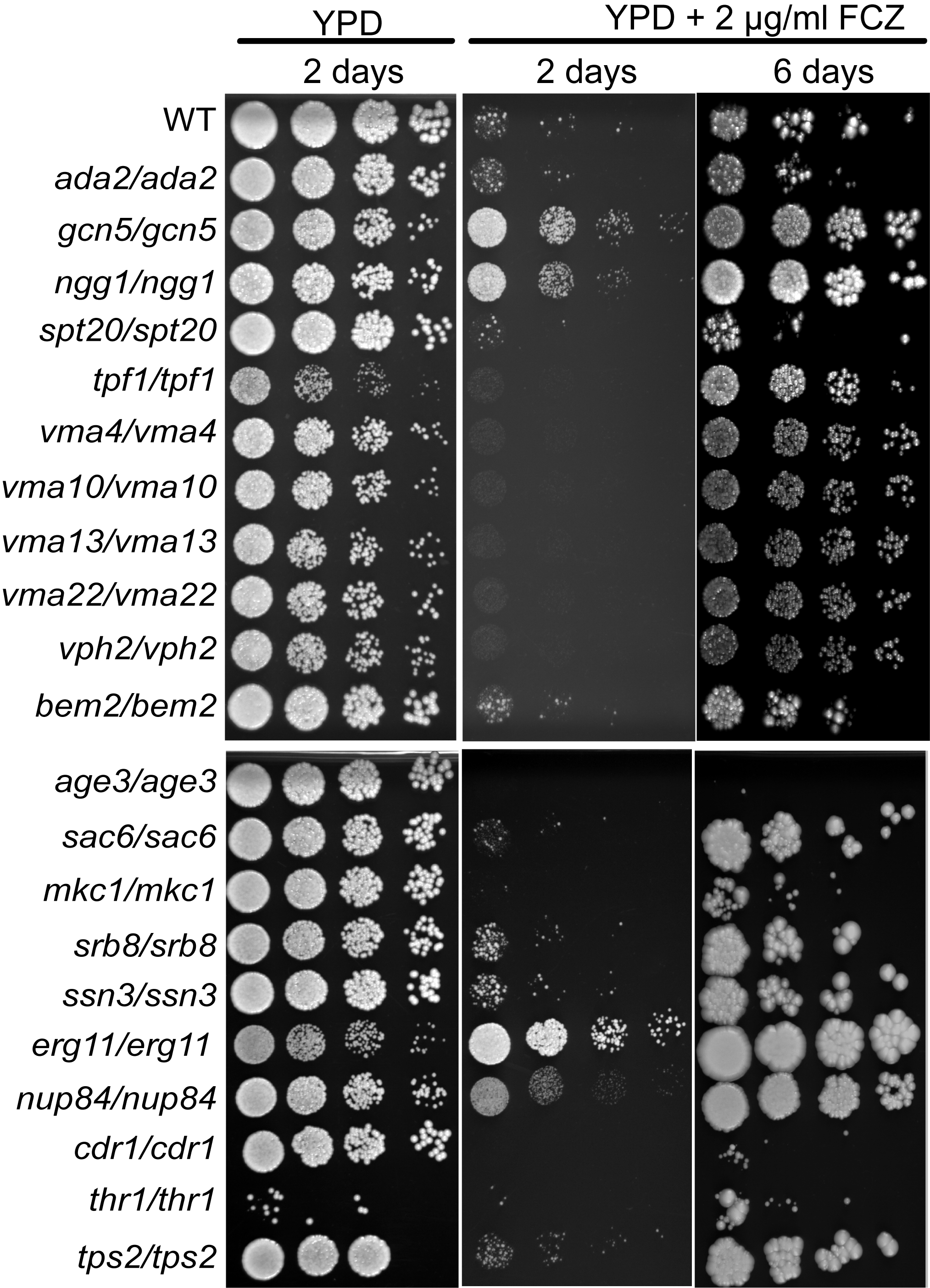

Supplement: Figure S1 — Phenotypes of the predicted FCZ-cidal genes in C. albicans as determined on rich media containing FCZ. Five-fold serial dilutions starting with an overnight culture diluted to OD600 of 0.1 was spotted (2 µl) on YPD or YPD + 2 µg/ml FCZ. Plates were incubated at 30°C for the time indicated. (5.05 MB TIF) [file ppat.1000753.s001.tif]

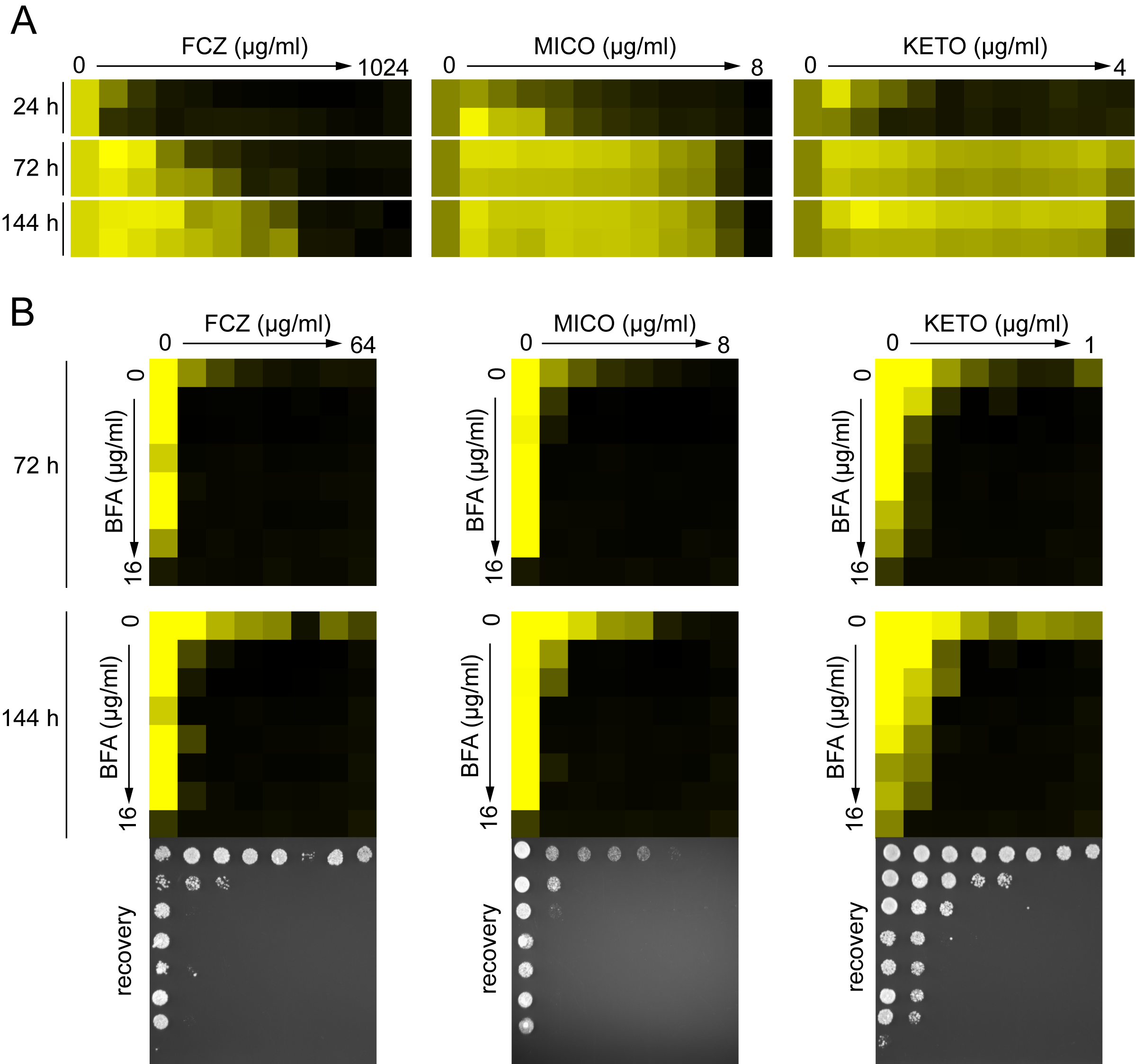

Supplement: Figure S2 — BFA synergy with different azoles in synthetically defined RPMI media at 30°C. (A) Prior to synergy testing, C. albicans WT strain was tested for drug sensitivity, which was measured over time on day one (24 hours), day three (72 hours) and day six (144 hours), respectively. Data was analyzed as in Figure 1A. (B) Optical densities of dose-matrix titration assays were measured on day three (72 hours) or day six (144 hours), respectively. Additionally, spot assays were done on day six. Except for media, assays were performed and analyzed as in Figure 1A and 3B. (2.15 MB TIF) [file ppat.1000753.s002.tif]

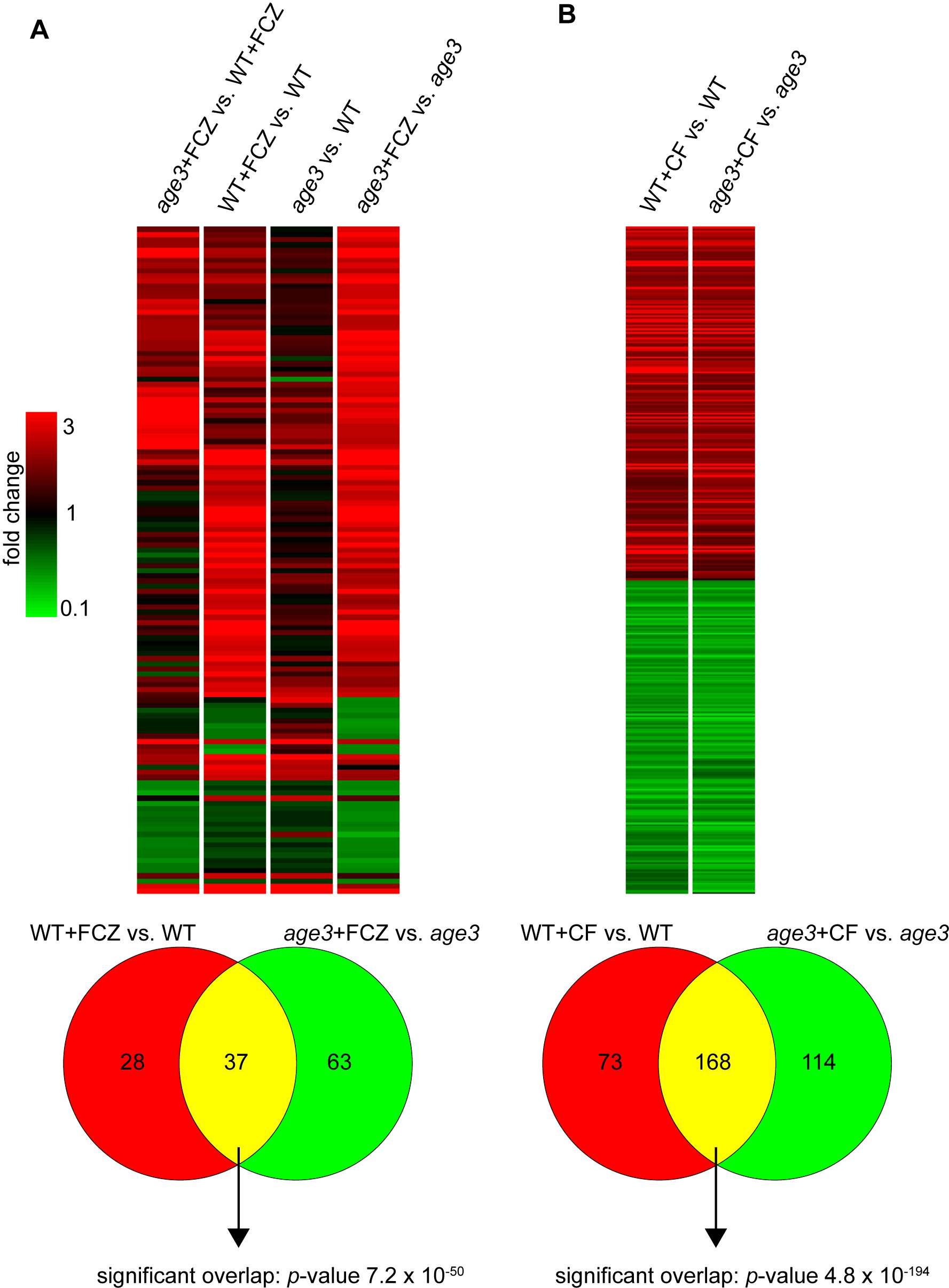

Supplement: Figure S3 — Core transcriptional responses to FCZ or CF are not significantly affected in the absence of AGE3. (A) Transcriptional analysis under FCZ treatment. Significantly regulated genes (>2 fold change, p-value <0.05) were selected when WT was treated with FCZ (WT+FCZ vs. WT) and combined with significantly regulated genes when age3 cells were FCZ treated (age3+FCZ vs. age3) to build a cluster tree (top). The same gene list was used to visualize in the Venn diagram (bottom) a significant overlap of core FCZ-responsive genes. (B) Transcriptional analysis under CF treatment. Gene lists were selected in the same way as described for the FCZ treatment to build a cluster tree. The Venn diagram illustrates that there is a significant overlap of core CF responsive genes. Tables S4, S5, S6, S7 and S8 list exact transcript changes for all significantly regulated genes used in the FCZ and CF analysis. (0.89 MB TIF) [file ppat.1000753.s003.tif]

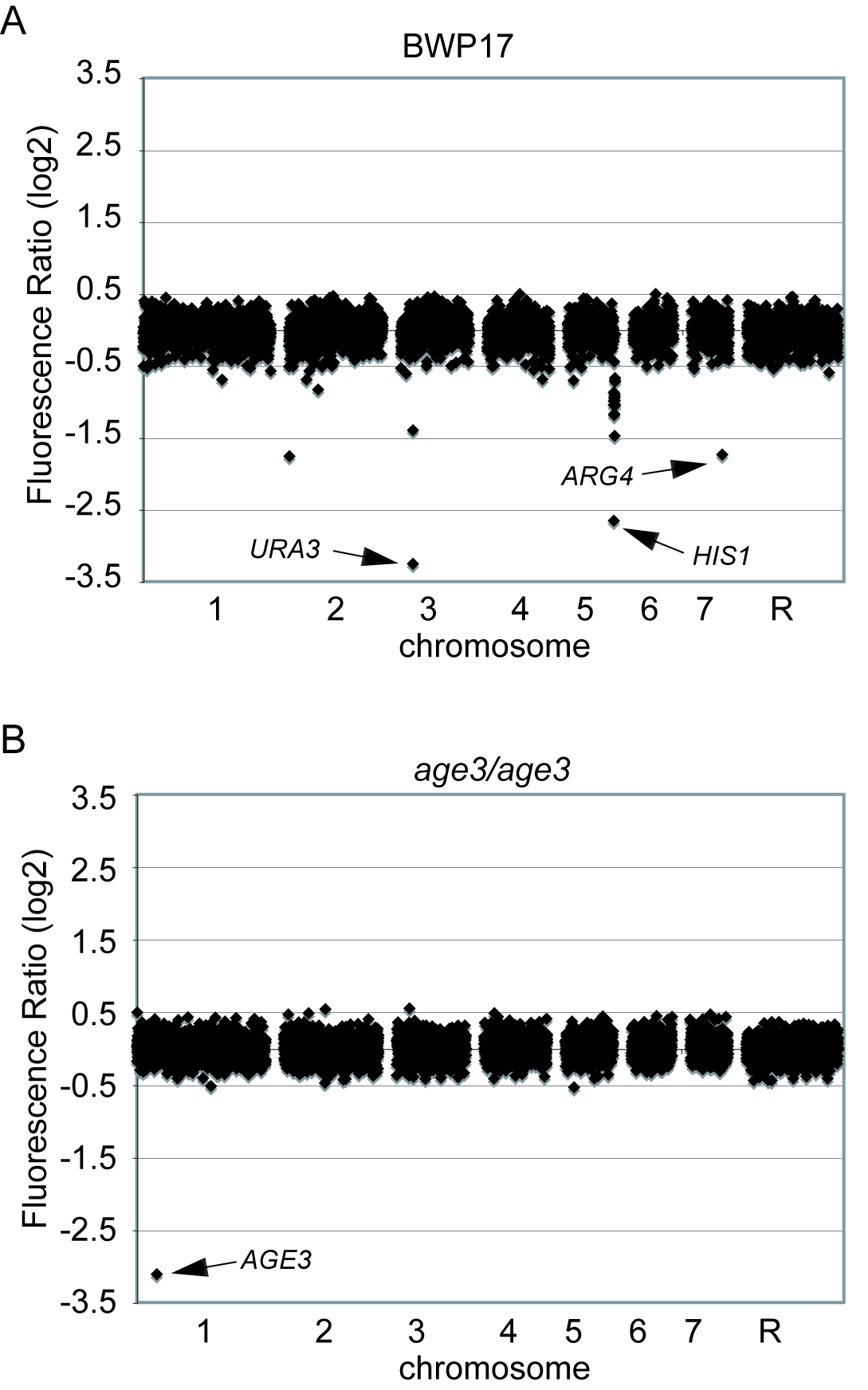

Supplement: Figure S4 — No aneuploidies were detected in the AGE3 deletion mutant by CGH analysis. Cy3 labeled genomic DNA from either age3 mutants or strain BWP17 was hybridized to DNA microarrays with Cy5 labeled genomic DNA from the reference strain SC5314. Shown are plots of CGH (comparative genome hybridization) analyses, where the y-axis shows the log2 fluorescence ratio of the mutant strains versus SC5314 and the x-axis shows all chromosomes (1 to R). A single black rhombus represents the log2 fluorescence ratio plotted as a function of its position in the C. albicans' assembly 21. In this representation, a 1.5-fold increase in fluorescence ratio (i.e. 3 chromosome copies versus 2) equals a log2 ratio of ∼0.58. (A) CGH shows the known loss of one end of chromosome 5 in strain BWP17. This strain is also auxotroph for URA3, HIS1, ARG4. (B) The prototrophic age3 mutant does not have any chromosomal rearrangements as determined by CGH. (7.62 MB TIF) [file ppat.1000753.s004.tif]
